# Nicotine-Free E-Cigarettes Might Promote Tobacco Smoking Reduction Better Than Nicotine Delivery Devices: Results of a Double-Blind Randomized Controlled Trial at 1 Year

**DOI:** 10.3390/curroncol29110676

**Published:** 2022-11-11

**Authors:** Claudio Lucchiari, Marianna Masiero, Ketti Mazzocco, Giulia Veronesi, Patrick Maisonneuve, Costantino Jemos, Emanuela Omodeo Salè, Stefania Spina, Raffaella Bertolotti, Derna Busacchio, Gabriella Pravettoni

**Affiliations:** 1 Department of Philosophy, University of Milan, 20122 Milan, Italy; claudio.lucchiari@unimi.it; 2 Department of Oncology and Hemato-Oncology, University of Milan, 20122 Milan, Italy; ketti.mazzocco@unimi.it (K.M.); gabriella.pravettoni@unimi.it (G.P.); 3 Applied Research Division for Cognitive and Psychological Science, IEO European Institute of Oncology, IRCCS, 20141 Milan, Italy; derna.busacchio@ieo.it; 4 Faculty of Medicine and Surgery, Vita-Salute San Raffaele University, 20132 Milan, Italy; veronesi.giulia@hsr.it; 5 Division of Thoracic Surgery, IRCCS San Raffaele Scientific Institute, 20132 Milan, Italy; 6 Division of Epidemiology and Biostatistics, IEO European Institute of Oncology, IRCCS, 20141 Milan, Italy; patrick.maisonneuve@ieo.it; 7 Division of Pharmacy, IEO European Institute of Oncology, IRCCS, 20141 Milan, Italy; costantino.jemos@ieo.it (C.J.); eomodeo@ieo.it (E.O.S.); 8 Division of Thoracic and General Surgery, Humanitas Research Hospital, 20089 Milan, Italy; stefania.spina@humanitas.it; 9 Division of Thoracic Surgery, IEO European Institute of Oncology, IRCCS, 20141 Milan, Italy; raffaella.bertolotti@ieo.it

**Keywords:** smoking, e-cigarette, personality, decision-making, cancer screening

## Abstract

The purpose of the present study was to determine whether the use of e-cigarettes to aid in quitting contributed to the increase in the pulmonary health of chronic smokers. The efficacy of e-cigarettes to support a successful smoking cessation attempt was also investigated. A total of 210 smokers (78 women) were enrolled in a screening program for the early detection of lung cancer and distributed in three arms: nicotine e-cigarette plus support, nicotine-free e-cigarette plus support, and support. Results showed that participants in the nicotine e-cigarette arm had a significant and fast decrease in daily cigarettes, but that later they resume smoking more than the other two groups. Conversely, participants in the other two arms showed similar daily consumption at the two evaluation points. Among abstinent participants, only 12.5% reported cough, while 48% of current smokers had pulmonary symptoms. Our study suggests that, in the long run, the use of a nicotine-free liquid may favor reducing smoking and could be considered a good option in a clinical setting.

## 1. Introduction

The public debate on the effectiveness and safety of e-cigarettes is currently ongoing [1,2] and has involved public health stakeholders as well as citizens around the world. A definitive international consensus should be reached in order to design a set of clinical recommendations for healthcare professionals who daily work with high risk-patients who continue smoking [3]. At the moment, there is a noteworthy lack of consistent findings about the effectiveness of e-cigarettes, especially in the long run, to support smoking interruption attempts [2,4], while health consequences remain controversial [3,5,6,7,8]. Walker and colleagues (2020) observed that nicotine e-cigarettes increased smoking abstinence compared with nicotine-free cigarettes after 6 months [9]. Nevertheless, the cessation rate at the end of the whole study was low in both groups (9% versus 4%). Further, in a large study named Population Assessment of Tobacco and Health Study (PATH study), the use of e-cigarettes led to similar abstinence rates (at months 12 and 24) compared to other cessation aids, such as transdermal nicotine patches, nicotine gum, and nicotine lozenges [2]. Similarly, Hajek and colleagues (2019) found that e-cigarettes were more effective in achieving 12 months of abstinence than nicotine replacement therapy (NRT) [10]. In particular, at 1 year, the abstinence rate was higher for participants that used e-cigarettes compared with participants that used other NRTs (18% versus 9.9%). Furthermore, the e-cigarette group reported greater improvement in pulmonary health from baseline to 52 weeks than the participants using NRT. Other studies reported that e-cigarette was positively associated with short-term (around 30 days) abstinence [2,11], but also to an increased relapse rate [12]. Farsalinos and colleagues (2020) stated that the real impact of the e-cigarette on smokers’ behavior and the safety of e-cigarettes should be evaluated taking into account both the e-cigarettes use and the smoking cessation period. They argued that intermittent use may only have marginal effects on the smoking status at 1 year and 1–3 years, while continuous e-cigarette use is positively associated with smoking cessation success [1]. Another study based on the E3 Trial (Evaluating the Efficacy of e-Cigarette Use for Smoking Cessation) [13] found that nicotine e-cigarettes combined with counseling increased abstinence at month 3 compared with counseling alone (21.9 versus 9.1%), but these results are only partially preserved at month 6 (17.2% versus 9.9%). The authors suggested that nicotine-free cigarettes may help smokers to cope better with behavioral and social aspects of tobacco dependence [13].

Concerning the potential health consequences of the habitual use of e-cigarettes, there is controversial evidence regarding short-term and long-term toxicity [5,14]. Kenkel and colleagues [15] examined the association between nicotine e-cigarettes and long-term respiratory diseases (mainly chronic obstructive pulmonary disease). Authors found in the never-tobacco smokers group no evidence that current or past e-cigarette use is associated with an increased risk of respiratory disease. Instead, they observed that former e-cigarette users and never-tobacco smokers had a reduced risk of respiratory disease. Overall, they reported a relatively low rate (about 4.5%) of pulmonary disease in the e-cigarette group. Hajejk and colleagues (2019) found only symptoms such as throat and mouth irritation [10]. Cobb and colleagues [16] stressed the potential advantages of cigarette cessation in the short term, observing decreased expired carbon monoxide and urinary cotinine levels when e-cigarettes were used exclusively. However, the dual use of cigarettes and e-cigarettes in this study did not reduce cigarettes smoked or expired carbon monoxide compared to tobacco cigarettes. These results are in line with other studies [17,18] suggesting that dual use can initially result in a reduction in cigarettes per day and related toxicant exposures. However, other studies associated dual users with impaired cardiopulmonary functions [19,20]. More research on dual-use effects on health is clearly needed [15,17,18]. Gordon and colleagues [14] also suggested that controlled clinical trials should be designed and implemented in order to provide a more comprehensive understanding of the mechanisms related to e-cigarette use and safety.

The primary outcome of the present trial was to assess the impact (at months 3, 6, and 12) of an e-cigarette program to reduce smoking-related respiratory symptoms (cough, breath shortness, catarrh) as a consequence of reduced tobacco cigarette consumption. The secondary outcomes included the assessment of the success rate of smoking cessation attempts and daily smoking reduction in the three arms (nicotine e-cigarette, nicotine-free e-cigarette, and controls), and the monitoring of safety and toxicity during the study in arms 1 and 2. As a general aim, we wanted to test the possibility to exploit the psycho-social characteristic of the screening context, often considered a teachable moment, to extend its benefits by providing help to stop cigarette smoking, and then reducing the participants’ lung cancer risk.

The current work reports data after 1 year [21]. Our previous publications described the complete protocol and results after 3 months and after 6 months [3,21,22].

## 2. Materials and Methods

### 2.1. The Study Design

The current study is a double-blind, randomized, controlled trial. Participants were enrolled at the IEO within the COSMOS II (Continuous Observation of SMOking Subjects) screening program for the early detection of lung cancer [23]. The study has been approved by the Ethical committees of the University of Milan and the European Institute of Oncology and was registered as a clinical trial (Registration code: NCT02422914). The research protocol and procedures followed the principles stated in the Declaration of Helsinki (59th WMA General Assembly, Seoul, 2008). Full details of the study protocol were published elsewhere [21]

### 2.2. Procedure

A sample of 210 smokers with a mean age of 62.8 (SD = 4.58) have been enrolled. All participants were chronic smokers, that is they have smoked at least 10 or more cigarettes per day for a time of 10 years or more [21]. Participants were distributed in three arms. Arm 1 (*n* = 70): participants received a treatment based on the use of nicotine e-cigarettes (an e-cigarette kit and 12 10-mL liquid cartridges (8 mg/mL nicotine concentration). Arm 2 (*n* = 70): participants were delivered a treatment based on the use of nicotine free e-cigarettes (an e-cigarette kit and 12 10-mL liquid cartridges similar to the one provided in Arm 1 but with no nicotine). Finally, in Arm 3 (n_70): participants received psychological counseling. Participants in Arms 1 and 2 were instructed to use the e-cigarette ad libitum for a period of 1-week since the enrollment day. Thereafter, participants had to use the e-cigarette for three months. Three assessment points were set at months 3, 6, and 12. 

Participants met the following inclusion criteria:

- To be involved in the COSMOS II study;
- To be chronic smokers (have smoked at least ten cigarettes a day for the past 10 years);
- To have a motivational score higher than 10 and not be treated at a smoking center.

Exclusion Criteria:

- Symptomatic cardiovascular disease;
- Symptomatic severe respiratory disease;
- Regular psychotropic medication use;
- Current or past history of alcohol abuse;
- Use of smokeless tobacco or NRT.

**Figure 1:**
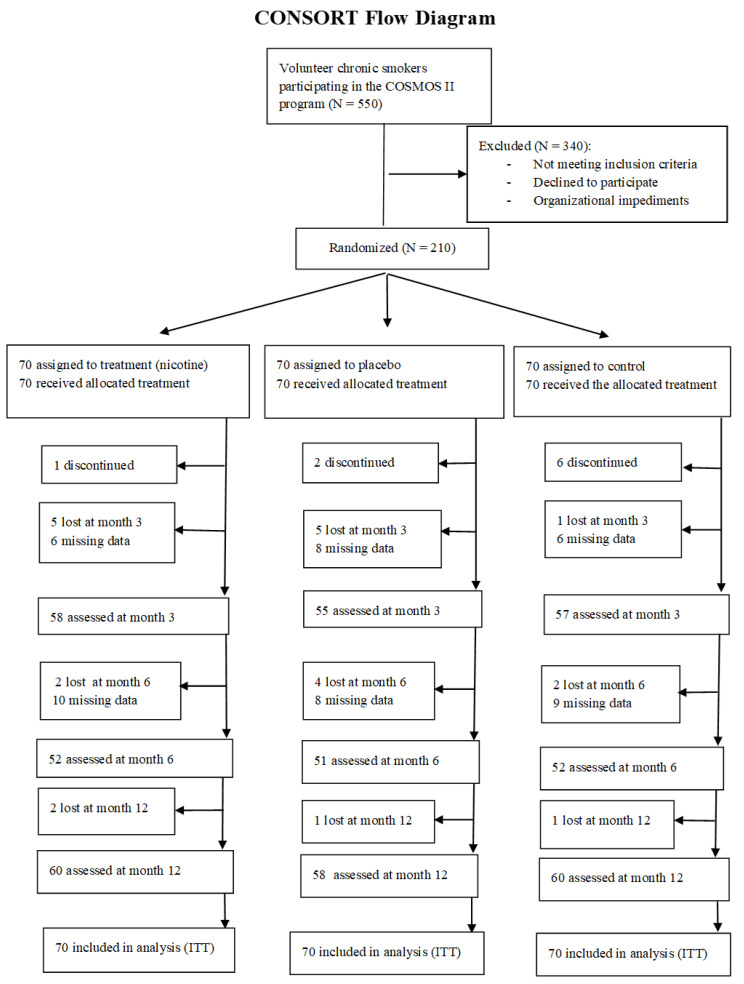
CONSORT Flow Diagram.

Considering the difficulty and the duration of the study, we decided to also maintain in the study participants that have not participated in all assessment points (Figure 1).

### 2.3. Instruments

To evaluate the pulmonary health, smoking behaviors, and psychological features of participants, we used both objectives and self-reported measures. 

Expired carbon monoxide (eCO) was measured using the Micro+™ Smokerlyzer^®^ (Bedfont Scientific Ltd.), which has less than 5% H2 cross-sensitivity [3,21]. ECO has been evaluated at months 6 and 12 by CO level. A value lower or equal to 7 ppm was considered to indicate a non-smoking status. 

*Fagerstrom Test for Nicotine Dependence (FTND)*: This self-reported questionnaire includes 6 items to assess the nicotine dependence level and it ranges 0–10, with higher scores indicating stronger dependence. It is a robust measure and it was proved reliable also in the Italian version we used [24].

*Motivational questionnaire*: it is a 4-item self-report questionnaire that measures smokers’ motivation to quit [25]. The questionnaire outcome classifies smokers into 4 motivational categories: 4–6 = low; 7–10 = middle; 11–14 = high; 15–19 = very high.

*Hospital Anxiety and Depression Scale (HADS)*: it includes 2 subscales (7 items each) to assess depression and anxiety [26]. The Italian version has been validated as a reliable scale [27,28]. 

*Leicester Cough Questionnaire (LCQ)*: it includes 19-items to assess how chronic cough impact one’s quality of life. It ranges 3 to 21, with better quality of life associated with higher scores [29,30].

*Respiratory symptoms*. Pulmonary health and e-cigarettes related adverse events were assessed by the use of a set of ad hoc items and checklists and then discussed through clinical interviews. Quality data about e-cigarettes use and experience was also collected. 

### 2.4. Statistical Analysis

The initial sample was sized to ensure the statistical power needed to test primary and secondary aims of the study [21]. Consistently, the reduction for each participant has been calculated taking into account the difference between the number of cigarettes smoked per day at the baseline and at 1-year. Changes in smoking status and frequency of respiratory symptoms were evaluated by the use of Chi-squared test. Participants were included into three groups based on their smoking status (Abstinent, Reduced consumption or No change). We defined abstinence as self-reported complete absence of tobacco cigarette use over the previous month. Thus, the abstinent group included who reported continuous abstinence over the previous month. This status was also controlled at month 6 and 12 by the use of eCO level, that must be under or equal 7 ppm. The reduction group included smokers that reported at least a 20% decrease in daily tobacco consumption compared to the baseline. All the other participants were considered current smokers with no status change. An Intention-to-treat analysis (ITT) was used. Mix-designed ANCOVA tests were used to evaluate significant changes in CO, Dependence Level, LCQ, and HADS. SPSS package (version 26.0, IBM, Chicago, IL, USA, 2019) was used. 

## 3. Results

### 3.1. Sample Characteristics

Due to drop-out and missing data, at month 12, the study sample included 178 participants: 60 in Arm 1 (Nicotine e-cigarette; 24 women and 38 men), 58 in Arm 2 (Nicotine-free e-cigarette group; 21 women and 37 men), and 60 in Arm 3 (Control group; 20 women and 40 men). See Table 1 for further details.

### 3.2. Primary Outcome Assessment: Improvement in Pulmonary Health

**Figure 2:**
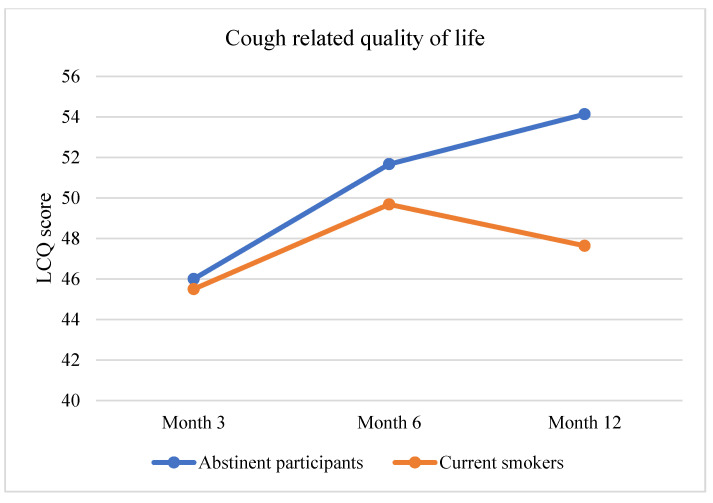
Change in Cough related quality of life as measured by LCQ throughout the study.

**Figure 3:**
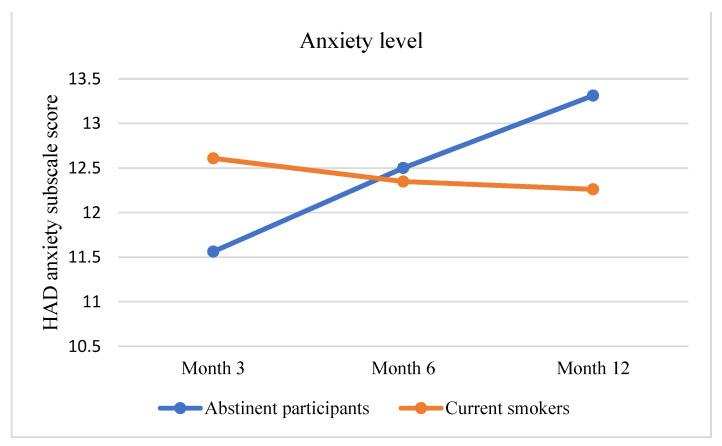
Change in anxiety level as measured by HAD subscale throughout the study.

Considering all participants, only 12.5% of abstinent participants reported cough versus about 48% of current smokers, while about 61% of abstinent participants reported episodes of breathlessness in the previous 6 months, versus about 80% of current smokers (in Table 2). Mixed-design ANCOVAs were run to evaluate changes in cough-related quality of life (LCQ), anxiety, and depression levels (HADS). Abstinent participants were found to report a significant increase in anxiety symptoms (F = 7.072, *p* = 0.001), while LCQ changes signaled an improvement in their cough-related quality of life (F = 3.373, *p* = 0.039) (see Figure 2 and Figure 3).

Burning throat decreased from 6 to 12 months, which may be considered the only relevant symptom potentially related to the habitual use of e-cigarettes (Table 3).

### 3.3. Secondary Outcome: Reduction of Tobacco Smoking

The number of participants who stopped smoking after 12 months by the study arm is reported in Table 4. The two groups that used e-cigarettes included more abstinent participants than control. However, we did not find significant differences between arms (*p* = 0.722). Furthermore, independently of the research arm, at month 12, about 17% of current smokers reported using also nicotine e-cigarettes while about 30% of abstinent participants reported using only electronic devices with e-liquids containing nicotine

**Figure 4:**
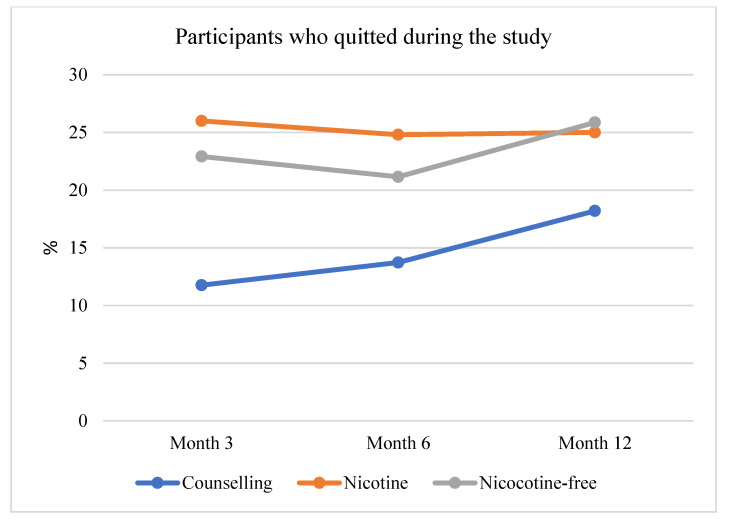
Percentage of quitters during the study by arm.

Considering the whole study, participants in arm 1 showed the highest quitters’ rate at month 3, while in the following evaluation points the number of abstinent smokers was reduced. A different trend has been revealed in arm 2 and arm 3. In particular, participants in arm 2 showed an increased rate between month 6 and month 12 (Figure 4).

To assess smoking reduction, we ran three mixed-design ANCOVAs taking the evaluation point (two levels, months 6 and 12) as the within-subjects factor, the study arm (3 levels, arm 1, arm 2, and arm 3) as the between-subjects factor, and the daily smoking baseline as the covariate. Results revealed that participants in arm 1 had a significant increase in daily cigarette smoking with respect to the other two arms (F = 3.979, *p* = 0.022). Indeed, at month 12, smokers in arm 1 smoked a mean of 16.18 tobacco cigarettes (S.D. = 1.251) versus a mean of 11.40 (S.D. = 6.4) cigarettes smoked at month 6. Conversely, participants in the other two arms showed similar daily consumption at the two evaluation points (14.655 vs. 13.711 in arm 2 and 13.560 vs. 13.933 in arm 3) where no statistical differences were detected. The other two ANCOVAs were on exhaled CO and on FTND scores, which did not show any significant differences.

Finally, we ran a correlational analysis to test if some baseline values were correlated with the daily cigarettes smoked at month 12. The variables that significantly correlated with the final daily consumption were the baseline dependence level measured by the FTND (r = 0.383, *p* < 0.0001), the number of daily cigarettes smoked at the beginning of the study (r = 0.326, *p* < 0.0001), and the anxiety level as measured by the HADs subscale (r =0.171, *p* = 0.46).

## 4. Discussion

This randomized clinical trial analyzed the efficacy of e-cigarettes in improving pulmonary health (primary aim) and, secondary, in supporting chronic smokers enrolled in a screening program [3,21,22]. Considering the primary aim, our results showed that after 12 months all abstinent participants, independently of the arm, had a substantial improvement in pulmonary health. More specifically, they reported improvement in general LCQ score indicating a better health-related quality of life due to a reduction of cough. Concerning the secondary aim, participants who vaped nicotine e-cigarettes included a higher number of quitters at month 3, but then this group experienced an increased tendency to relapse at month 6 and 12. Otherwise, participants in Arm 2 (nicotine free e-cigarettes) and Arm 3 (counselling alone) showed an opposite trend. In particular, participants who vaped nicotine-free e-cigarettes experienced a higher reduction in daily cigarette consumption. We argue that this might suggest that nicotine delivery devices might be disadvantageous for smokers with time, since it could increase the physical dependence. Furthermore, this phenomenon might be associated with a dual pattern use. Indeed, accruing evidence stressed that smokers using e-cigarettes frequently tend to combine e-cigarettes and traditional cigarettes with time, thus increasing the nicotine consumption and dependence [2]. Consistently, in our sample we found that about the 17% of current smokers used both cigarettes and nicotine e-cigarettes.

Even if previous studies have reported conflicting results about e-cigarettes-related adverse events, in the current study, we did not observe any severe side-effects during the whole study. The main side-effect reported was burning throat, while cough, nausea, and stomachache were rarely present. Furthermore, all side-effects decreased from month 6 to month 12. In this context, our results are coherent with previous studies reporting only mild side-effects such as throat and mouth irritation [10], which may be linked to ingredients of liquids, since e-cigarettes users and even non-users who passively vaped frequently report similar symptoms [31]. However, our results support the safety of e-cigarettes used in a clinical controlled setting, where participants were constantly monitored and received support for any issues or doubts they might have. Nevertheless, considering the controversial evidence reported by several studies [5,6,8,32,33], further research is needed to better define the risks of the e-cigarettes used habitually by both, as well as never smokers and non-users in order to provide guidelines for safe use. This is particularly important in settings where e-cigarettes are meant as devices to aid quitting [3,4]. As recently suggested by Gordon and colleagues [14], future controlled clinical trials should be specially designed and implemented in order to provide a more comprehensive understanding of the mechanisms behind e-cigarette use and health outcomes.

An important outcome of our study is related to the reduction in daily cigarettes. Participants included in the nicotine e-cigarettes arm had a significant increase in daily cigarette consumption compared to other participants from month 6 to month 12. This increase nullified the reduction achieved in the first 6 months of the study. On the contrary, participants in Arm 2 and 3 maintained their tobacco daily consumption from month 6 to month 12. Looking at the smoking reduction in the short term (month 3), we can observe a fast decrease in daily cigarettes smoked in Arm 1 and Arm 2 with the highest impact of nicotine cigarettes [3,22]. In our study, participants were involved in a 3-month program. In this period, participants in Arm 1 were asked to use e-cigarettes with a specific dosage and frequency and they were supported by telephone counseling. Thus, the obtained results must be linked to this setting that showed to be superior in terms of smoking reduction than the one of arm 3 based only on counseling. It is likely that the nicotine delivered by the e-cigarette suppressed withdrawal symptoms, thus increasing subjective responsivity to the treatment [34] beyond the behavioral effects that one might expect also by the use of nicotine-free tools.

At the end of the 3-month program, participants were not supported anymore and were free to continue with e-cigarettes, completely stopping, switching to traditional or dual smoking. At month 6, participants in Arm 1 showed an increased average of daily cigarettes, while other participants’ smoking showed a plateau. Coherently, Ebbert and colleagues [35] found that nicotine e-cigarettes initially reduce traditional smoking, but also maintain nicotine dependence at a high level, thus increasing the probability of relapse and/or of increasing tobacco smoking in the long run. Furthermore, we found that initial values for the dependence level, number of daily cigarettes, and anxiety are positively associated with the number of daily cigarettes at 1 year. This suggests that physical and psychological variables might be important modulators of the success of a quitting attempt. The presence of correlations suggests that they should be included in future studies and that it is difficult to provide clear evidence of e-cigarettes’ efficacy as anti-smoking tools without considering several methodological, contextual, and individual concerns.

In conclusion, the whole project’s outcomes may be considered sound support to the idea that e-cigarettes are safe and valid tools to help smokers to stop or reduce tobacco consumption. However, many other variables beyond the choice of a specific nicotine delivery system play important roles in an anti-smoking setting. A growing number of studies highlighted the key role of psychological intervention in tobacco smoking cessation programs involving both for first- and second-line treatments [4,36,37].

Several studies have shown the importance of including some form of help for smokers to quit smoking or at least reduce the number of daily cigarettes smoked in cancer screening procedures [38]. It is known, in fact, that a reduction in the number of cigarettes can reduce the risk of developing lung cancer, albeit significantly less than complete cessation [39]. Our study evaluated testing the hypothesis that the use of electronic cigarettes by people with a long smoking history and, therefore, at high risk of developing lung cancer may promote a substantial reduction in cigarettes smoked, thereby reducing the risk of lung cancer. Specifically, our intention was to show how in a particular sample of the smoking population, including those who voluntarily undergo a screening procedure periodically being part of a long-term project, cigarette use can be particularly beneficial. In fact, these smokers, despite not having been able to quit smoking continuously during their lifetime, show that they are concerned about their risk of developing cancer and that they need to control this risk in some way [40]. The electronic cigarette for these people could be an effective way to further control this risk without completely abandoning a well-established behavior that is felt to be indispensable. At present, studies on the relationship between electronic cigarettes and lung cancer development are few, while assessments about related risks are controversial [41]. However, some studies show that replacing cigarettes with e-cigarettes reduces the presence of substances considered carcinogenic after a few weeks of full or partial replacement [42]. We hope that the use of an anti-smoking protocol, such as the one we tested, will become a common practice for lung cancer screenings, so as to offer participants an actionable perspective to reduce smoking behavior and better control, in this way, their risk of developing cancer or to limit its worst consequences. Moreover, the use of nicotine-free e-cigarettes may be beneficial, since the effect might be maintained over time without sustaining or increasing the nicotine dependence level.

Finally, we observed that a significant number of current smokers started to use both traditional and electronic cigarettes, showing a dual use that could have later negative effects. Furthermore, when deciding to use e-cigarettes to support quitting, comorbidities such as asthma should be considered in order to prevent severe adverse events. This is valid also for nicotine-free e-cigarettes. The outcomes and the several issues raised by this study and others strongly suggest that more and more focused research is needed to define and share policies for stakeholders and clinical guidelines for healthcare professionals to properly approach the huge problem of tobacco smoking.

## 5. Limitations

Regarding the study limitations, the sample size and characteristics can be discussed. As already reported in previous publications [3,21,22], inferences about the effectiveness of electronic cigarettes on quitting must be considered in light of the study design and its statistical power, that was calculated to detect change in pulmonary health. Second, we experienced missing data during each of the evaluation points and this fact reduced the statistical power of other analyses. Third, our sample included only people older than 55 years with a long history of smoking who decided to take part in a screening program and with a high motivation to stop smoking. Furthermore, since we opted for a quite low nicotine, our data may be partially different from what reported by other studies using higher dosage, e.g., the one of Cobb and colleagues [16].

## Figures and Tables

**Table 1:** Descriptive statistics: participant’s main characteristics at month 12.

Variable	Arm 1		Arm 2		Arm 3	
	M	SD	M	SD	M	SD
Smoking starting age	17.55	3.77	16.90	3.58	17.76	3.68
Daily cigarettes	16.18	7.23	13.71	7.22	13.93	7.20
e-CO (ppm value)	13.11	7.01	12.21	7.17	14.63	6.99
Dependence Level	4.48	1.69	4.20	1.80	4.67	1.72
Motivational Level	12.00	3.30	13.16	3.09	12.42	4.11
Anxiety (HADs)	12.17	2.20	12.45	2.37	12.12	2.24
Depression (HADs)	9.13	1.57	8.90	1.81	8.32	1.37
LCQ	48.83	7.16	46.14	6.12	50.30	5.47

**Table 2:** Respiratory symptoms at month 12.

		Cough %	Catarrh %	Breathlessness %	Bronchitis %
Nicotine e-cigarette					
	Abstinent participants	13.3	10.3	61.1	9.1
	Current smokers	51.1	47.1	80.0	15.2
Nicotine-free e-cigarette					
	Abstinent participants	13.3	13.0	63.0	9.6
	Current smokers	37.2	40.0	72.1	10.0
Control					
	Abstinent participants	10.0	13.0	59.5	13.1
	Current smokers	40.0	42.6	82.0	8.1
All participants					
	Abstinent participants	12.5	11.8	61.2	10.2
	Current smokers	48.0	43.9	80.1	10.5

**Table 3:** Side-effects. Here we describe participants’ subjective reports about e-cigarettes’ side-effects. Answers were collected through an interview by health personnel concerning the last 6 months.

	Burning Throat	Cough	Nausea	Headache	Insomnia	Stomachache	Confusion
*Month 6*							
Nicotine e-cigarette group	15.9%	5.8%	5.8%	-	1.4%	4.3%	1.4%
Nicotine-free e-cigarette group	5.6%	2.8%	7.0%	1.4%	-	4.2%	-
*Month 12*							
Nicotine e-cigarette group	7.8%	3.8%	4.1%	-	-	4.3%	-
Nicotine-free e-cigarette group	1.2%	-	5.0%	-	-	4.5%	-

**Table 4:** Numbers of current smokers and abstinent participants at month 12 by groups.

	Abstinent Participants	Current Smokers	Total
Arm 1 (Nicotine e-cigarette)	15 (25.0%)	45 (75.0%)	60
Arm 2 (Nicotine-free e-cigarette)	15 (25.9%)	43 (74.1%)	58
Arm 3 (Control)	10 (16.7%)	50 (83.3%)	60
Tot	40 (22.5%)	138 (77.5%)	178

## Data Availability

The data presented in this study are available on request from the corresponding author.
